# The incidence of cytomegalovirus infection after deceased-donor kidney transplantation from hepatitis-C antibody positive donors to hepatitis-C antibody negative recipients

**DOI:** 10.1080/0886022X.2020.1835675

**Published:** 2020-10-26

**Authors:** Masahiko Yazawa, Tibor Fülöp, Orsolya Cseprekal, Manish Talwar, Vasanthi Balaraman, Anshul Bhalla, Ambreen Azhar, Csaba P. Kovesdy, James D. Eason, Miklos Z. Molnar

**Affiliations:** a James D. Eason Transplant Institute, Methodist University Hospital, Memphis, TN, USA; b Division of Transplant Surgery, Department of Surgery, University of Tennessee Health Science Center, Memphis, TN, USA; c Division of Nephrology and Hypertension, Department of Internal Medicine, St. Marianna University School of Medicine, Kawasaki, Japan; d Division of Nephrology, Department of Medicine, Medical University of South Carolina, Charleston, SC, USA; e Medicine Service, Ralph H. Johnson VA Medical Center, Charleston, SC, USA; f Department of Transplantation and Surgery, Semmelweis University, Budapest, Hungary; g Division of Nephrology, Department of Medicine, University of Tennessee Health Science Center, Memphis, TN, USA; h Nephrology Section, Memphis Veterans Affairs Medical Center, Memphis, TN, USA

**Keywords:** Cytomegalovirus infection, end-stage kidney disease, direct-acting antiviral agents, hepatitis C, kidney transplantation, real-word experience

## Abstract

**Background:**

Deceased-donor kidney transplantation (KT) from hepatitis C (HCV)-infected donors into HCV-uninfected recipients (HCV D+/R−) could become standard care in the near future. However, HCV viral replication by viral transmission might lead to a higher incidence of cytomegalovirus (CMV) infection in these recipients.

**Methods:**

A national-registry-based retrospective cohort study was conducted using the Scientific Registry of Transplant Recipients (SRTR) data set. We assessed the incidence of CMV infection in HCV antibody (Ab) negative recipients receiving kidneys from HCV Ab positive (HCVAb D+/R−) and negative (HCVAb D−/R−) donors. The risk of CMV infection was analyzed by Cox regression analysis in a propensity score (PS) matched-cohort of HCVAb D+/R− (*n* = 950) versus HCVAb D−/R− (*n* = 950). Sensitivity analysis was also conducted in the entire cohort (*n* = 181 082).

**Results:**

The mean age at baseline was 54 years, 75% were male, and 55% of the patients were African American in PS-matched cohort. Compared to the HCVAb D−/R − patients, recipients with HCVAb D+/R − showed identical probability for the incidence of CMV infection (Hazard Ratio (HR) = 1.00, 95% Confidence Interval (CI): 0.82–1.22). In the sensitivity analysis, compared to the HCVAb D−/R − patients, the HCVAb D+/R − group had a significantly lower risk of CMV infection in the unadjusted analysis (HR = 0.75, 95%CI: 0.65–0.85), while this risk difference disappeared after the adjusted analysis (HR = 0.99, 95%CI: 0.87–1.14).

**Conclusion:**

The incidence of CMV infection was similar in recipients who received HCVAb D + and HCVAb D − KT. Further studies are needed to assess this association in KT from HCV nucleic acid positive donors.

## Introduction

Not only strictly designed clinical trials [1–3], but also real-world experience outside of clinical trials [4] have strongly advocated for the utility and safety of deceased-donor kidney transplantation (KT) from hepatitis-C (HCV)-infected donors to HCV-uninfected recipients (HCV D+/R−), followed by the administration of direct-acting antiviral agents (DAA). In an era plagued by both organ shortage and a crisis of opioid-abuse-related deaths, this strategy may offer an opportunity of increasing the donor pool and decreasing the organ discard rate [5–8]. According to data from national registry data analyses, KT from HCV D+/R − fared similarly or better than KT from HCV D−/R − KT recipients during the initial six to twelve months, matched for each recipient’s and donor’s characteristics, including KDPI [7,9]. Furthermore, this new strategy of donation (HCV D+/R−) followed by DAA treatment has accomplished a 100% sustained virologic response (SVR) by week 12, irrespective of viral-load, genotypes, or the timing of DAA administration after KT [1–4,8]. The aggressive utilization of HCV-donor kidneys would reduce the excess mortality and morbidity experienced by waitlisted patients with end-stage kidney disease (ESKD) [10] and save medical costs, owing to a shortened waiting time [11]. This new strategy of using HCV-infected donor kidneys for transplantation into uninfected recipients might indeed become the new standard in industrialized societies.

Despite excellent overall clinical outcomes reported from well-designed clinical trials [1–3], there were a few reports of unfavorable consequences of HCV infected kidney transplantation into uninfected recipients, such as a higher risk of BK polyoma and cytomegalovirus (CMV) viremia [4]. We previously documented that the incidence rate of CMV viremia after D+/R − KT was approximately double compared to the expected incidence in non-HCV-related KT with appropriate CMV prophylaxis [4,12,13]. However, it is not known whether HCV infection directly stimulates CMV reactivation/infection or contributes to immunosuppression. Indeed, HCV viral replication might theoretically create a milieu for secondary viral infections by enhancing pro-inflammatory and profibrotic processes in BK virus infection [14] and by the modification of the natural killer (NK) cells’ subset in CMV infection [15,16]. In real-world experience, the approval of DAA by a third-party payer may take a considerable amount of time, that is, our former study reported a median duration of 76 days for starting DAA after KT [4]. This relatively longer delay preceding DAA administration may enable an interim massive HCV replication and a higher incidence of CMV infection [5]. Furthermore, although CMV infection is now easily controlled by prophylaxis treatment and, once CMV infection has occurred, it will confirm worse patient and kidney allograft outcomes [17,18].

Our study hypothesis was that transplanting patients across a hepatitis-C discordant status, those with HCV D+/R − transplantation are more likely to experience a higher incidence of CMV virus infection, compared to those undergoing HCV D−/R − KT. To test this hypothesis, we conducted a propensity score (PS) matched cohort study using the Scientific Registry of Transplant Recipients (SRTR) data set.

## Materials and methods

### Cohort definition and data source

This study used data from the Scientific Registry of Transplant Recipients (SRTR). The datasets generated during and/or analyzed during the current study are available in the SRTR repository (www.srtr.org). This national-registry-based retrospective cohort study was conducted from a publicly available United States SRTR data set. The SRTR data system includes data on all donors, wait-listed candidates, and transplant recipients in the US, submitted by the members of the Organ Procurement and Transplantation Network (OPTN). The Health Resources and Services Administration (HRSA), U.S. Department of Health and Human Services provides oversight to the activities of the OPTN and SRTR contractors [19]. Unfortunately, the outcomes of interest (CMV infection) have not been collected systematically after April 2015, while our original exposure of interest [nucleic acid test (NAT) results of donor HCV] was reported in the SRTR database only after April 1st, 2015. Therefore, we decided to use a cohort, which was transplanted before April 2015 together with the donors’ HCVAb-based definition for exposure.

**Figure 1.**
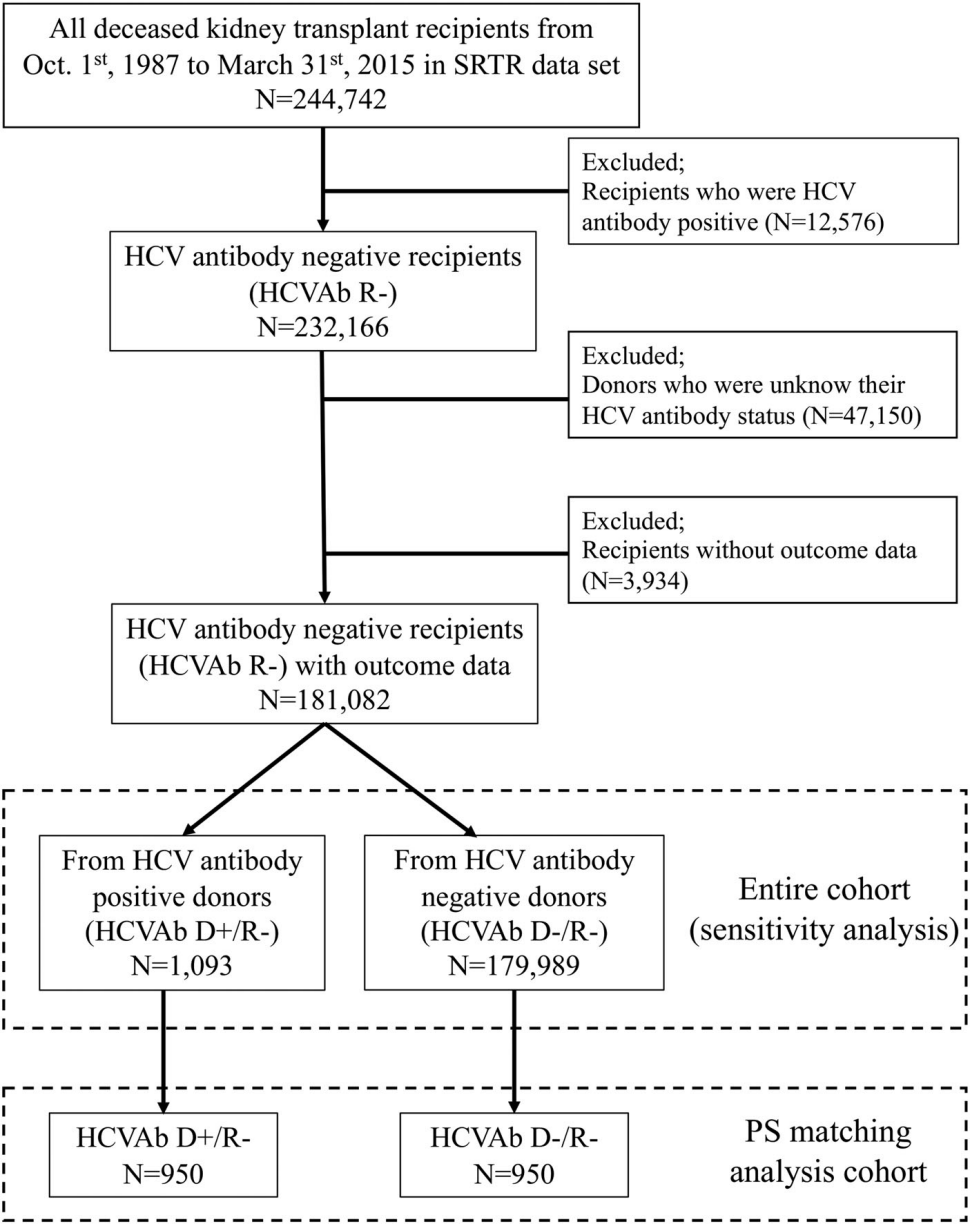
Flow chart of patient selection. Abbreviations. SRTR: Scientific Registry of Transplant Recipients; HCV: hepatitis C virus; HCVAb: hepatitis C virus antibody; HCVAb D+/R−: kidney transplantation from hepatitis-C-antibody-positive donor into negative recipient; HCVAb D−/R−: kidney transplantation from hepatitis-C-antibody-negative donor into negative recipient.

The baseline cohorts contained 244742  deceased-kidney-transplant recipients from October 1st, 1987 to March 31st, 2015. Of those, we excluded non-eligible recipients according to the following criteria: HCVAb positive recipients (*n* = 12 576), donors with an unknown HCVAb status (*n* = 47 150) and those without outcome data (*n* = 3934). After extracting the participants based on the above exclusion criteria, 181 082 HCVAb-negative recipients (HCVAb R−) with outcome data were included in the analysis. For the analysis, we divided the recipients into two groups based on the donors’ HCVAb seropositivity; one group received kidneys from HCVAb-positive donors (HCVAb D+/R−, *n* = 1093) and the other from HCVAb-negative donors (HCVAb D−/R−, *n* = 179 989). For our main analysis, we created a propensity-score-matched cohort including 950 HCVAb D+/R − and 950 HCVAb D−/R − recipients (Figure 1).

### The definition of the exposure and control groups

Exposure was defined based on donor HCVAb status. The exposure group was defined as recipients of kidneys from HCVAb-positive donors (HCVAb D+), while the control group’s donors were HCVAb negative (HCVAb D−). Unfortunately, records of the donors’ HCV nucleic acid test (NAT) results, which could prove the active infection of HCV and data about CMV infections on the national registry dataset, were not available in the same time period. Therefore, we used the serostatus of the HCV antibody (HCVAb) as a potential surrogate for active viral replication instead of the HCV nucleic acid test (NAT) assay. The exact numbers and proportions of both exposure and control groups are shown in Figure 1.

### Outcome assessment

The primary endpoint was the incidence of first CMV infection. The definition of first CMV infection was based on the captured first treatment for CMV after transplantation. The treatment was defined as using any of the following medications: Immune Globulin Intravenous (CytoGam^®^), valganciclovir, ganciclovir, and valacyclovir. However, the data set did not clearly distinguish the actual treatment from prophylaxis therapy for CMV infection. Therefore, we created an algorithmic classification for CMV infection based on risk and its captured medical treatment. Briefly, we divided the CMV risk classification into three categories based on CMV IgG before KT in both donors and recipients. The low-risk group was defined as the combination of CMV IgG negativity both in donors (CMV IgG D−) and recipients (CMV IgG R−), namely CMV IgG D−/R−. The intermediate-risk group was defined as either CMV IgG D−/R+, or D+/R+. The high-risk group was defined as CMV IgG D+/R− (Supplementary Figure 1). According to these three categories and the usual prophylaxis strategy, those who were administrated valacyclovir within 90 days after KT in the low-risk group and valacyclovir or valganciclovir within 90 days after KT in the intermediate-risk group and 180 days after KT in the high-risk group were assigned as prophylaxis treatment during prophylactic period. The administration of any of these drugs after the above-mentioned prophylactic periods was counted as evidence for the ‘first CMV infection’, which is defined as an outcome event in this study (Supplementary Figure 1).

### Covariates

The following information has been collected from the SRTR database about our recipients: age, sex, race, body mass index (BMI), induction therapy including anti-thymocyte globulin (ATG), any calcineurin inhibitors (CNI) and mycophenolate acids (MPA) at discharge, history of KT and organ transplantation, a history of delayed graft function (DGF) defined as a need for at least one dialysis session within 1 week after transplantation, results of the calculated panel reactive antibody (cPRA), and the numbers of human leukocyte antigen (HLA) mismatches.

The following information has been extracted from the SRTR database about deceased donors: age, sex, race, BMI, history of diabetes (DM), cause of death, donation after cardiac death, and serum creatine before donation. CMV risk classification, as mentioned above, was a critical confounder and was used as a matching covariate (Supplementary Figure 1).

### Statistical analysis

Baseline characteristics were presented in the HCVAb D+/R − and HCVAb D−/R − groups as mean ± standard deviation (SD) or median and interquartile range (IQR) for continuous variables, and numbers and percentages (%) for categorical variables, as appropriate. Differences between groups were analyzed by student *t*-tests or the Mann–Whitney test for continuous variables and the chi-square test for categorical variables. Standard differences that were compared between the HCVAb D+/R − and HCVAb D−/R − groups were also described in both the entire cohort and the PS matched cohort.

For the survival analysis in both the main (PS matched) and sensitivity (entire cohort) analyses, the start of the observational period was the date of KT, and all recipients were followed-up until the date of CMV incidence or any of the following censoring events: death, allograft loss or end of follow-up (1 April 2015), whichever came first.

For the main analysis, the propensity score (PS) method was used to account for the confounding effects arising from differences in the participants’ baseline characteristics in those who were assigned as HCVAb D+/R − and HCVAb D−/R−. First, to detect the covariates likely to influence the probability of HCVAb D+/R−, a logistic regression analysis was conducted (presented in Supplemental Table 1). Subsequently, variables associated with HCVAb D+/R − were identified and used for calculating PSs. We used the ‘psmatch2’ command in STATA to generate the 1:1 PS matched cohort using the nearest neighbor matching without replacement (Figure 1 and Table 1). The following variables were used for the logistic regression model to create the PS: recipients’ age, sex, race, induction therapy, CNI, type of prior organ transplantation if any, DGF and HLA mismatches; donor’s age, sex, race, DM, donation after cardiac death (DCD), cause of death, and CMV risk classification. The distribution of PSs in both the HCVAb D+/R − and HCVAb D−/R − groups before and after matching are shown in Supplementary Figure 2.

**Table 1.** Baseline characteristics of the entire cohort and the propensity matching cohort compared between HCVAb D+/R − and HCVAb D−/R−.

Baseline characteristics	Entire cohort, *n* = 181 082					PS matching cohort *n* = 1900			
	HCVAb D+/R−, *n* = 1093	HCVAb D−/R−, *n* = 179 989	*p*-Value*	Standardized difference	Total missingNo.	HCVAb D+/R−, *n* = 950	HCVAb D−/R−, *n* = 950	*p*-Value^†^	Standardized difference
Recipient information									
Age, years, mean ± SD	53.8 ± 11.7	48.7 ± 15.4	<0.001	0.381	0	54.0 ± 11.6	54.0 ± 13.3	0.892	0.006
Sex, male, *n* (%)	823 (75.3)	107 581 (59.8)	<0.001	−0.340	0	716 (75.4)	718 (75.6)	0.915	0.005
BMI, kg/m^2^, mean ± SD	26.9 ± 5.2	27.2 ± 5.7	0.107		24 341	27.0 ± 5.2	27.2 ± 5.4	0.377	
Race, *n* (%)			<0.001	0.253	4			0.860	0
Caucasian	468 (42.8)	115 235 (64.0)				396 (41.7)	385 (40.5)		
African American	587 (53.7)	51 537 (28.6)				520 (54.7)	533 (56.1)		
Asian	28 (2.6)	9979 (5.5)				26 (32.7)	28 (3.0)		
Native American	4 (0.4)	1949 (1.1)				3 (0.3)	2 (0.2)		
Pacific Islander	3 (0.3)	899 (0.5)				2 (0.2)	1 (0.1)		
Multiracial	3 (0.3)	386 (0.2)				3 (0.3)	1 (0.1)		
Induction therapy, *n* (%)			<0.001	−0.100	11 376			0.946	−0.009
Non-induction	354 (36.2)	41 438 (24.6)				342 (36.1)	337 (35.5)		
ATG	256 (26.2)	68 849 (40.8)				250 (26.3)	251 (26.4)		
Alemtuzumab	51 (5.2)	12 838 (7.6)				51 (5.4)	50 (5.3)		
IL-2 receptor blocker	232 (23.7)	36 346 (21.5)				224 (23.6)	237 (25.0)		
OKT3	85 (8.7)	9257 (5.5)				82 (8.6)	75 (7.9)		
CNI use at discharge, *n* (%)	1015 (95.9)	168 693 (95.1)	0.269	0.015	2659	908 (95.6)	919 (96.7)	0.189	−0.060
MPA use at discharge, *n* (%)	770 (72.7)	144 636 (81.6)	<0.001		2659	727 (76.5)	751 (79.1)	0.185	
Previous any organ transplantation, *n* (%)	192 (17.6)	24 783 (13.8)	<0.001	0.077	55	158 (16.6)	149 (15.7)	0.575	0.026
Previous kidney transplantation, *n* (%)	137 (12.5)	22 877 (12.7)	0.862		0	116 (12.2)	132 (13.9)	0.276	
HLA mismatch, *n* (%)			<0.001		779			0.541	
0	15 (1.4)	22 221 (12.4)				13 (1.4)	17 (1.8)		
1	16 (1.5)	3358 (1.9)				9 (1.0)	8 (0.8)		
2	37 (3.4)	11 601 (6.5)				33 (3.5)	25 (2.6)		
3	140 (12.9)	28 262 (15.8)				118 (12.4)	94 (9.9)		
4	284 (26.1)	45 295 (25.3)				248 (26.1)	255 (26.8)		
5	377 (34.7)	46 455 (25.9)				335 (35.3)	353 (37.2)		
6	218 (20.1)	22 024 (12.3)				194 (20.4)	198 (20.8)		
Total HLA mismatches, n, mean ± SD	4.5 ± 1.3	3.7 ± 1.8	<0.001	0.518	779	4.5 ± 1.2	4.5 ± 1.2	0.320	−0.046
cPRA, %, median (IQR)	0 (0, 2)	0 (0, 5)	<0.001		4840	0 (0, 2)	0 (0, 3)	0.037	
Delayed graft function, *n* (%)	285 (26.2)	42 317 (23.6)	0.044	0.079	310	256 (27.0)	258 (27.2)	0.918	−0.005
Donor information									
Age, years, mean ± SD	39.7 ± 10.9	36.8 ± 17.0	<0.001	0.205	0	39.8 ± 11.0	39.4 ± 16.9	0.531	0.029
Sex, male, *n* (%)	735 (67.3)	107 546 (59.8)	<0.001	−0.147	0	635 (66.8)	629 (66.2)	0.771	−0.013
BMI, kg/m^2^, mean ± SD	25.4 ± 5.3	26.3 ± 6.4	<0.001		2311	25.4 ± 5.3	26.9 ± 6.3	<0.001	
Donor Race, *n* (%)			<0.001	0.041	51			0.825	−0.006
Caucasian	922 (84.4)	151 463 (84.2)				807 (85.0)	810 (85.3)		
African American	164 (15.0)	23 005 (12.8)				137 (14.4)	132 (13.9)		
Asian	7 (0.6)	3847 (2.1)				6 (0.6)	8 (0.8)		
Other	0	1623 (0.9)				0	0		
Donation after cardiac death, *n* (%)	34 (3.1)	15 558 (8.7)	<0.001	−0.237	47	32 (3.4)	37 (3.9)	0.540	−0.028
Cause of death, *n* (%)			0.007	0.016	19			0.887	−0.040
Anoxia	177 (16.2)	33 098 (18.4)				157 (16.5)	150 (15.8)		
Cerebrovascular/stroke	398 (36.5)	64 622 (35.9)				345 (36.3)	332 (35.0)		
Head trauma	498 (45.7)	76 774 (42.7)				435 (45.8)	453 (47.7)		
Central nerve system tumor	1 (0.1)	1331 (0.7)				1 (0.1)	2 (0.2)		
Other	17 (1.6)	4147 (2.3)				12 (1.3)	13 (1.4)		
Comorbidity-diabetes, *n* (%)	37 (3.5)	9842 (5.5)	0.004	−0.114	995	31 (3.3)	31 (3.3)	1.000	0
Serum creatinine before donation, mg/dL, mean ± SD	1.07 ± 1.16	1.13 ± 1.14	0.050		420	1.03 ± 0.88	1.21 ± 1.46	0.001	
Serum creatinin*e* > 1.5 mg/dL before donation, *n* (%)	97 (9.0)	24 490 (13.6)	<0.001		406	78 (8.3)	142 (15.0)	<0.001	
CMV risk classification			<0.001	0.392	0			0.817	0.011
Low-risk group, *n* (%)	43 (3.9)	18 382 (10.2)				42 (4.4)	38 (4.0)		
Intermediate-risk group, n (%)	473 (43.3)	92 312 (51.3)				439 (46.2)	445 (46.8)		
High-risk group, *n* (%)	105 (9.61)	26 782 (14.9)				95 (10.0)	105 (11.1)		
Unknown-risk group, *n* (%)	472 (43.2)	42 513 (23.6)				374 (39.4)	362 (38.1)		

Abbreviations. PS: propensity score; HCVAb: hepatitis-C antibody; HCVAb D+/R−: kidney transplantation from hepatitis-C-antibody-positive donor into negative recipient; HCVAb D−/R−: kidney transplantation from hepatitis-C-antibody-negative donor into negative recipient; No.: number; SD: standard deviation; BMI: body mass index; ATG: anti-thymocyte globulin; IL-2: interleukin 2; OKT3: anti-CD3 antibody; CNI: calcineurin inhibitor; MPA: mycophenolate acid: HLA: human leukocyte antigen; cPRA: calculated panel reactive antibody; IQR: interquartile range; CMV: cytomegalovirus.

Definitions. Low risk: CMV IgG D−/R−; intermediate risk: CMV IgG D−/R + or CMV IgG D+/R+; high risk: CMV IgG D+/R−.

*Compared between HCVAb D+/R − and HCVAb D−/R − in the entire cohort; ^†^Compared between HCVAb D+/R − and HCVAb D−/R − in the PS matching cohort.

*p*-Values for continuous variables with mean ± SD are results of *t*-test and with median (IQR) are result of the Mann–Whitney test, and categorical variables are chi-square test.

The association between the donors’ HCVAb status and the incidence of CMV infection was assessed using the Kaplan-Meier method with the Log-rank test and using Cox proportional hazard models. Since the PS matched cohort was already well-matched, the Cox regression analysis was not additionally adjusted for covariates. We performed additional subgroup analyses to assess the association between HCVAb status and the incidence of CMV infection in the following *a priori* defined groups: age (less than or equal to 55 versus greater than 55 years), sex, race (non-African American versus African American), induction therapy (no induction versus any induction therapy), prior organ transplantation, cPRA (0–80% versus greater than 80%), and DCD. Potential interactions were formally tested by including relevant interaction terms.

For the sensitivity analysis, the entire cohort was used to compare the HCVAb D+/R − and HCVAb D−/R − groups (Figure 1). The association between the donors’ HCVAb status and the incidence of CMV infection was assessed using the Kaplan–Meier method, the Log-rank test, and the unadjusted and adjusted Cox proportional hazard models. We adjusted for the following confounders: recipients’ age, sex, race, induction therapy, CNI, prior organ transplantation, DGF and HLA mismatches; donor’s age, sex, race, DM, DCD, cause of death, and CMV risk classification. A sub-group analysis was also conducted by the same stratification that we applied at the PS-matched analysis. Potential interactions were formally tested by including relevant interaction terms.

*P* values were two-sided and the significance level was set at less than 0.05 for all analyses. All analyses were conducted using STATA Version 13 (STATA Corporation, College Station, TX). This study was approved by the Institutional Review Committee of The University of Tennessee Health Science Center (18-05819-NHSR). All research was performed in accordance with relevant guidelines/regulations, and informed consent was waivered as the analysis was performed in a national de-identified dataset.

## Results

### Baseline characteristics of the entire and the PS matched cohorts

Table 1 shows the baseline characteristics of both the HCVAb D+/R − and HCVAb D−/R − groups in the entire and the PS matched cohorts. In the entire cohort, there were 1093 recipients with HCVAb D+/R− (0.6%) (Figure 1). The HCVAb D+/R − group was significantly older with a higher prevalence of male sex and African American descent, as well as a lower usage of ATG as induction therapy and MPA as maintenance therapy compared to the HCVAb D−/R − group. Based on the available data, the HCVAb D+/R − group had more recipients with a lower CMV risk compared to the HCVAb D−/R − group, but this difference disappeared after PS matching.

There were 1900 recipients included in the main analysis after PS matching (Figure 1). The mean age at KT was 54.9 years, the majority was male, and half of the recipients were African American. The prevalence of high risk according to the CMV risk classification was 10%. Most of the variables were well-balanced as shown in Table 1.

### The incidence of CMV infection after kidney transplantation in the PS matched cohort

**Figure 2.**
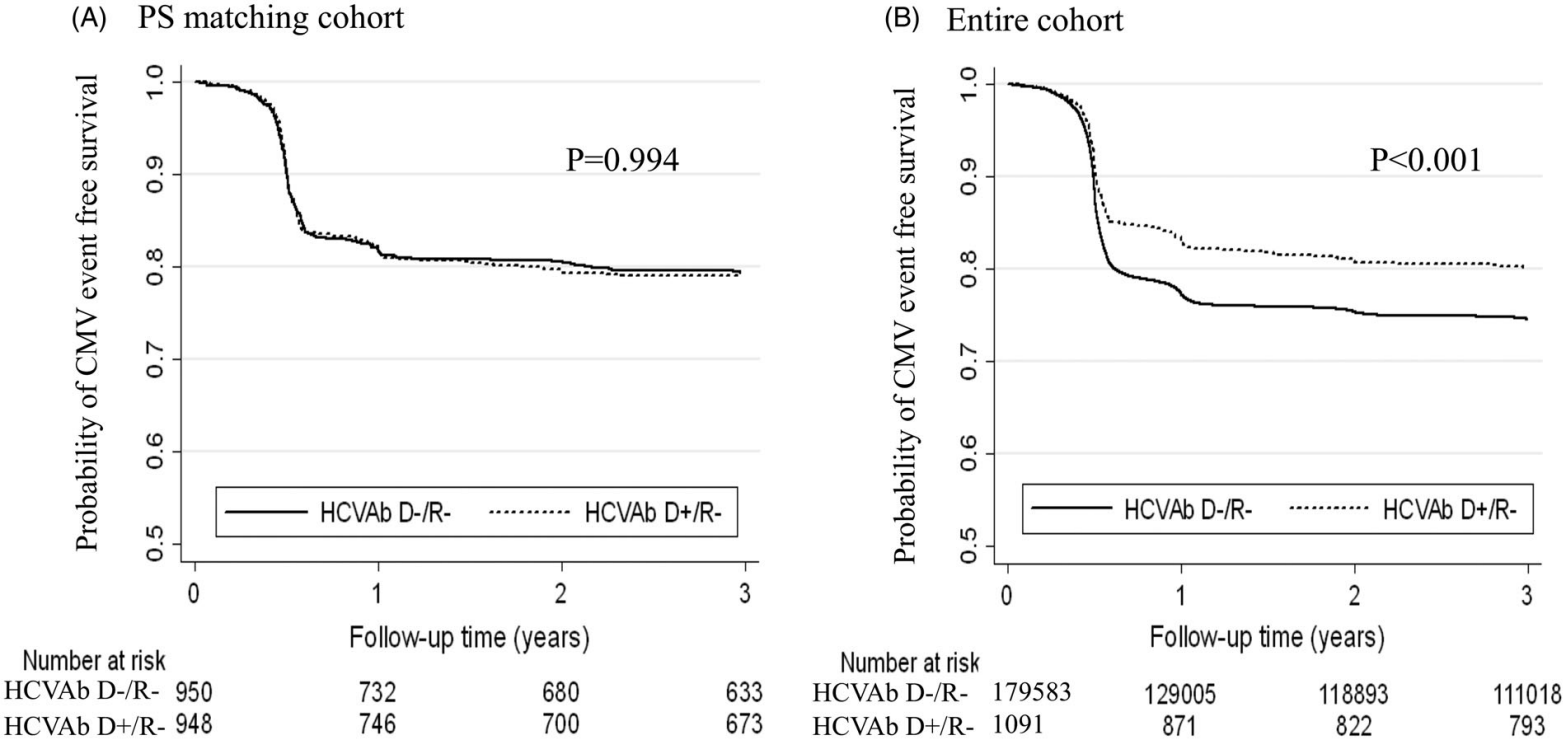
Kaplan–Meier curve for the probability of cytomegalovirus event free survival in PS matching cohort (panel A) and in the entire cohort (panel B) in the HCVAb D+/R − and HCVAb D−/R − groups. Abbreviations: PS: propensity score; HCVAb D+/R−: kidney transplantation from hepatitis-C-antibody-positive donor into negative recipient; HCVAb D−/R−: kidney transplantation from hepatitis-C-antibody-negative donor into negative recipient.

The median follow-up time was 8.6 (IQR: 1.5–15.8) years in the PS matched cohort. CMV infection occurred in 407 patients (incidence rate 23.6/1000 person-years, 95%CI: 21.4–26.0). The incidence rate was 22.8/1000 person-years (95%CI: 19.9–26.1) in the HCVAb D+/R − group and 24.5/1000 person-years (95%CI: 21.4–28.2) in the HCVAb D−/R − group (Figure 2(A), log-rank test *p* = 0.994). The HCVAb D+/R − group had a risk of CMV infection (HR = 1.00, 95%CI: 0.82–1.22) similar to the HCVAb D−/R − group (Table 2).

**Table 2.** Association between HCVAb D+/R − and CMV infection using the univariate and adjusted Cox proportional models.

	CMV infection		
PS matching cohort	HR	95%CI	*p-*Value
Univariate analysis			
HCVAb D+/R− (vs. HCVAb D−/R−)	1.00	0.82–1.22	0.994
Entire cohort			
Univariate analysis			
HCVAb D+/R− (vs. HCVAb D−/R−)	0.75	0.65–0.85	<0.001
Multivariate analysis			
HCVAb D+/R− (vs. HCVAb D−/R−)	0.99	0.87–1.14	0.935

Multivariate analysis in entire cohort was adjusted by recipient’s age, sex, race, induction therapy, use of calcineurin inhibitor, previous any type of transplantation, delayed graft function, HLA mismatch and donor’s age, sex, race, diabetes, donation after circulation death, cause of death, and CMV risk classification.

Abbreviations: HCVAb: Hepatitis C antibody; HCVAb D+/R−: Kidney transplantation from hepatitis-C-antibody-positive donor into negative recipient; HCVAb D−/R−: Kidney transplantation from hepatitis-C-antibody-negative donor into negative recipient; CMV: cytomegalovirus; HR: hazard ratio; 95%CI: 95% confidence interval; DSA: donor specific antibody.

### Sub-group analysis for the incidence of CMV infection in the PS matched cohort

**Figure 3.**
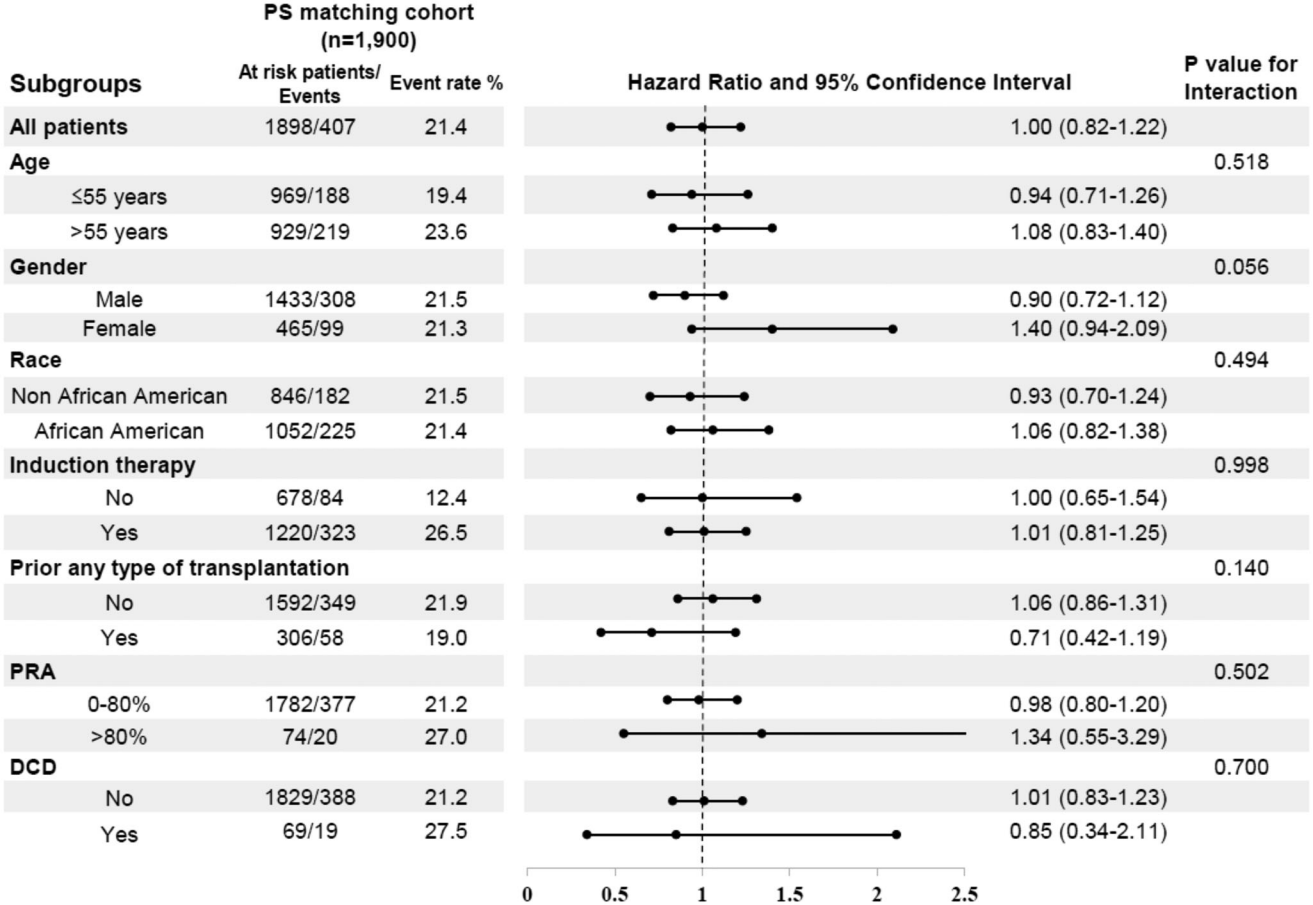
Association between HCVAb D+/R − and CMV infection in selected sub-group analyzed by Cox regression analysis among PS matching cohort. Abbreviations. PS: propensity score; cPRA: calculated panel-reacted antibody; DCD: donation after cardiac death.

Figure 3 shows the results of the subgroup analysis stratified by age, sex, race, induction therapy, prior organ transplantation, cPRA, and DCD. HCVAb D+/R − was not associated with CMV infection in any of the sub-groups.

### Sensitivity analysis for the incidence of CMV infection in the entire cohort

The median follow-up time was 6.0 (IQR: 0.7–13.6) years and CMV infection occurred in 46 020 patients (incidence rate: 33.4 cases/1000 person-year, 95%CI: 33.1–33.7) in the entire cohort. The incidence rate was 20.3/1000 person-years (95%CI: 17.8–23.1) in the HCVAb D+/R − group and 33.5/1000 person-years (95%CI: 33.2–33.8) in the HCVAb D−/R − group (Figure 2(B), Log-rank test *p* < 0.001). The HCVAb D+/R − group had a significantly lower risk of CMV infection in the unadjusted analysis (HR = 0.75, 95%CI: 0.65–0.85) compared to the HCVAb D−/R − group, whereas the HCVAb D+/R − group was not exposed to any significant risk of CMV infection in the adjusted analysis (HR = 0.99, 95%CI: 0.87–1.14) compared to the HCVAb D−/R − group (Table 2).

### Sub-group analysis or the incidence of CMV infection in the entire cohort

**Figure 4.**
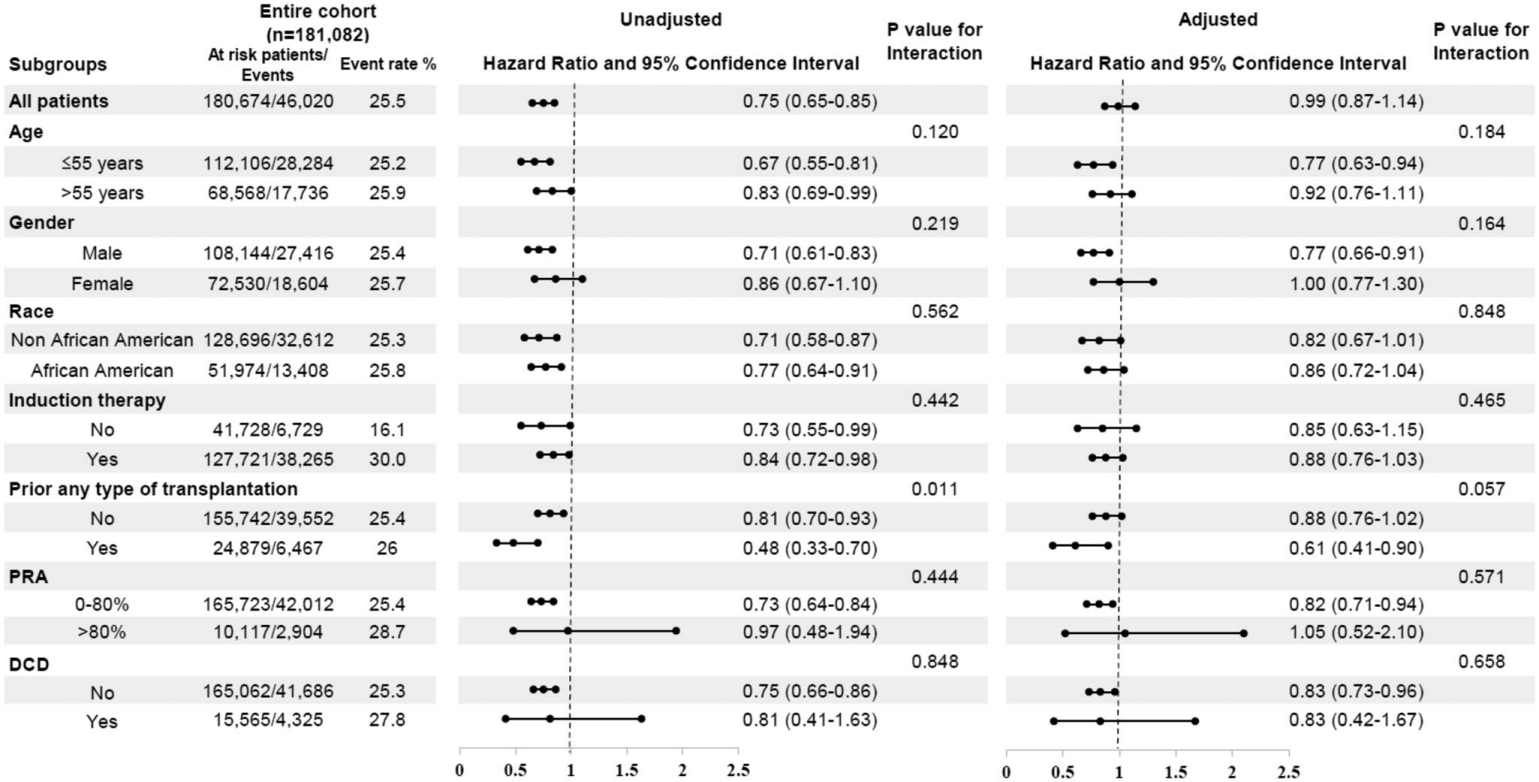
Association between HCVAb D+/R − and CMV infection in selected sub-group analyzed by unadjusted and adjusted Cox regression analysis among the entire cohort. Abbreviations. PS: propensity score; cPRA: calculated panel-reacted antibody; DCD: donation after cardiac death. Adjusted confounders were recipient’s age, sex, race, induction therapy, use of calcineurin inhibitor, previous organ of transplantation, delayed graft function, HLA mismatch and donor’s age, sex, race, diabetes, donation after circulation death, cause of death, and CMV risk classification.

Figure 4 shows the results of the unadjusted and adjusted sub-group analyses. In the unadjusted analysis, only the group with a history of organ transplantation had significant interaction, however, both hazard ratios indicated a lower risk of CMV infection. In the adjusted analysis, only younger age, male sex, a history of any organ transplantation, and non-DCD donors were significantly associated with a lower risk of CMV infection. However, no interaction existed in any of these sub-groups.

## Discussion

Contrary to our hypothesis, applying PS matching analysis and adjusted Cox regression analysis in the sensitivity analysis of the entire cohort in this national-registry-based cohort study showed a comparable incidence of first CMV infection between the HCVAb D+/R − and D−/R − groups. Moreover, subgroup analyses yielded similar outcomes. To the best of our knowledge, this is the first large, nationally representative study comparing the incidence of CMV infection between those with a potential for HCV transmission (HCVAb D+/R−) and those without. Altogether, these results provide cautious reassurance regarding the current strategy of accepting donations from HCV-infected deceased donors. However, additional qualifiers need to be considered when interpreting our results.

Previous data indicated potential pathophysiological connections between CMV and HCV virus infection in organ transplant recipients. It has long been known that CMV infection in liver transplant recipients due to HCV cirrhosis is strongly associated with HCV replication and a recurrence of HCV hepatitis and cirrhosis [20,21]. However, it is unknown whether HCV replication would have an effect on the risk of CMV disease in non-liver organ recipients through modification of the immune system. Some studies have not corroborated this association and exact mechanisms have not been well known, but CMV may confer an immunomodulatory effect via indirect effects and dysregulate specific cytokines against HCV replication [22]. Indeed, HCV infection *per se* can also promote conditions that are likely to reactivate both BK [14] and CMV infections via several mechanisms [15,16]. Chronic viral infections such as HCV and HIV alter natural killer (NK) cell subsets and impair the defensive ability against viral infections, including CMV [15,16].

When thinking about the association between HCV transmission and CMV reactivation/infection, we have to take into consideration whether DAA treatment is administered, as well as the duration between KT and the initiation of DAA. Delays with starting DAA might contribute to massive HCV replication and consequently might be associated with a higher incidence of CMV infection [4,5]. Our results are strictly applicable to the pre-DAA era as DAA treatment became available for kidney transplant recipients only after 2015 [23,24]. Further studies are needed to assess the association between HCV NAT + donor transplantation and CMV viremia risk in kidney and other solid organ transplant recipients.

Although this is a national-registry-based and adequately powered study, we should acknowledge its several limitations. First, the definition of exposure measurement is not precise due to the fact that we could not use the NAT results representing actual HCV infection. About one-third of the HCVAb + cases [25] are known to not represent real infected patients secondary to false-positive results, self-cleared, or post-HCV treatment status. In this regard, actual results might be interpreted as underestimation in the direction of either harm or benefit. Second, we were only able to use CMV treatment as outcome measurement instead of actual CMV viremia, therefore we likely underestimated the real incidence rate since we could not capture the actual incidence of CMV viremia or disease. To elucidate a proper association between HCV D+/R − and CMV infection, one would have to conduct a more specific cohort study using CMV viremia and disease as an outcome measure and HCV NAT results as an exposure. Third, this study was a retrospective cohort study. Ultimately, we could not clarify the causality between HCV transmission and the incidence of CMV infection. Fourth, we have recognized the immortal period until three to six months after KT due to the universal prophylaxis strategy shown in Figure 2.

In conclusion, the incidence of first CMV infection was similar in recipients who received HCVAb D + and HCVAb D − kidney transplantations. To further confirm these findings on this evolving topic, further studies using more rigorous exposure variables (HCV NAT results) and outcome criteria (CMV viremia and treatment) are strongly encouraged.

## Supplementary Material

Supplemental Material

Supplemental Material
