# Supplementary material for: The incidence of cytomegalovirus infection after deceased-donor kidney transplantation from hepatitis-C antibody positive donors to hepatitis-C antibody negative recipients
Source: Ren Fail. 2020 Oct 26;42(1):1083–92. doi: 10.1080/0886022X.2020.1835675 (PMC7594852; doi:10.1080/0886022X.2020.1835675)
Supplement: Supplemental Material [file IRNF_A_1835675_SM9832.pdf]

Supplementary Figure 1. Algorithm for the discrimination treatment from prophylactic treatment for CMV infection.

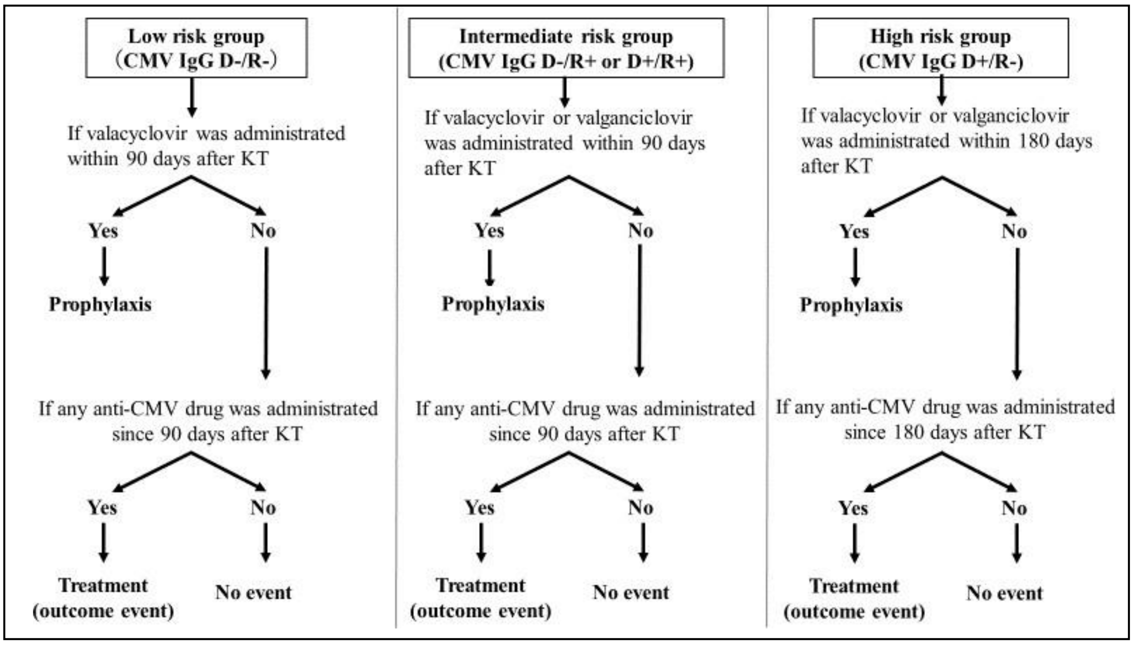

Abbreviations: CMV: Cytomegalovirus; IgG: Immunoglobulin G; KT: Kidney transplantation

Supplementary Figure 2. The distribution of the raw propensity scores in both HCVAb D+/R- and HCV D-/R- groups before and after propensity matching.

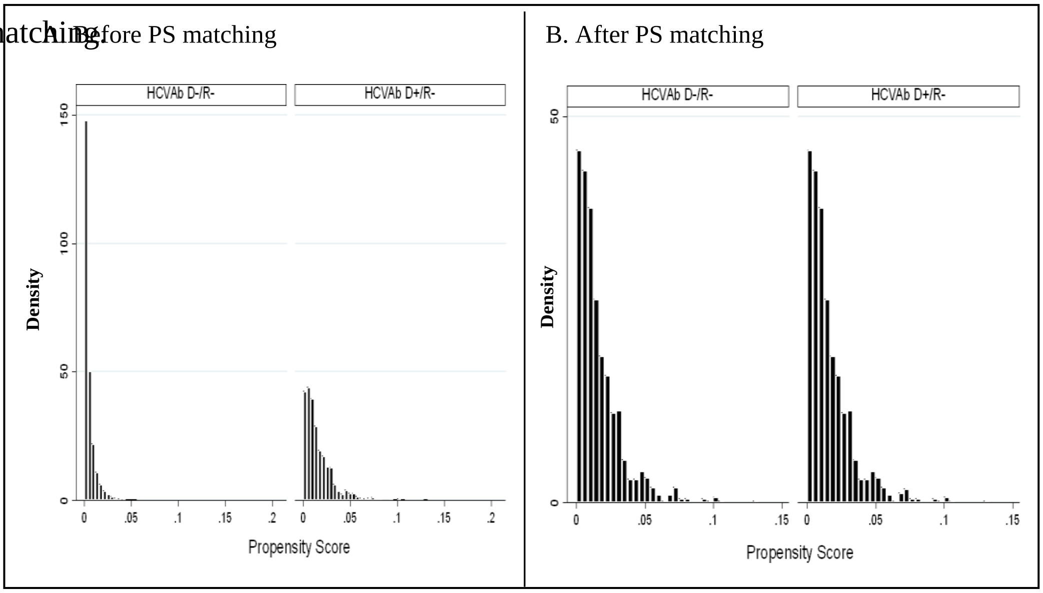

Abbreviations: PS: Propensity score; HCVAb: Hepatitis C virus antibody; HCVAb D+/R-: Kidney transplantation from hepatitis-C-antibody-positive donor into negative recipient; HCVAb D-/R-: Kidney transplantation from hepatitis-C-antibody-negative donor into negative recipient
