# Supplementary material for: The incidence of cytomegalovirus infection after deceased-donor kidney transplantation from hepatitis-C antibody positive donors to hepatitis-C antibody negative recipients
Source: Ren Fail. 2020 Oct 26;42(1):1083–92. doi: 10.1080/0886022X.2020.1835675 (PMC7594852; doi:10.1080/0886022X.2020.1835675)
Supplement: Supplemental Material [file IRNF_A_1835675_SM9829.pdf]

Supplementary Table 1. Probability of the kidney transplantation from HCV-antibody-positive donors (HCVAb D+) using logistic regression analysis.

Covariates	Odds ratio	95% CI	*p* value
**Recipient information**			
Age (+1 year)	1.04	1.03 to 1.04	<0.001
Sex (vs male)	0.52	0.45 to 0.60	<0.001
Race			
African American (vs Caucasian)	2.92	2.55 to 3.36	<0.001
Asian (vs Caucasian)	0.73	0.49 to 1.09	0.124
Native American (vs Caucasian)	0.48	0.15 to 1.49	0.203
Pacific Islander (vs Caucasian)	0.67	0.17 to 2.69	0.569
Multi-racial (vs Caucasian)	2.72	0.86 to 8.58	0.088
Induction therapy			
Alemtuzumab (vs non-induction therapy)	0.62	0.46 to 0.84	0.002
Thymoglobulin (vs non-induction therapy)	0.53	0.44 to 0.63	<0.001
IL-2 receptor blocker (vs non-induction therapy)	1.01	0.84 to 1.20	0.956
OKT3 (vs non-induction therapy)	0.81	0.63 to 1.05	0.108
CNI use at discharge (vs non-use)	1.18	0.86 to 1.61	0.309
Previous any type of transplantation (vs. absence)	2.02	1.69 to 2.41	<0.001
1 HLA mismatch (vs 0 HLA mismatch)	3.85	1.64 to 9.03	0.002
2 HLA mismatches (vs 0 HLA mismatch)	4.19	2.20 to 7.98	<0.001

3 HLA mismatches (vs 0 HLA mismatch)	6.21	3.50 to 11.0	<0.001
4 HLA mismatches (vs 0 HLA mismatch)	8.54	4.87 to 15.0	<0.001
5 HLA mismatches (vs 0 HLA mismatch)	11.7	6.70 to 20.5	<0.001
6 HLA mismatches (vs 0 HLA mismatch)	14.2	8.04 to 25.0	<0.001
Delayed graft function (vs. absence)	1.03	0.88 to 1.19	0.730
**Donor information**			
Age (+1 year)	1.01	1.00 to 1.01	<0.001
Sex (vs male)	0.73	0.64 to 0.85	<0.001
Race			
Asian (vs American Indian/Alaska native)	>100	N/A	0.981
African American (vs American Indian/Alaska native)	>100	N/A	0.979
Caucasian (vs American Indian/Alaska native)	>100	N/A	0.979
Comorbidity-diabetes (vs absence)	0.48	0.33 to 0.69	<0.001
Donation after circulatory death (vs absence)	0.36	0.25 to 0.52	<0.001
Cause of death			
Cerebrovascular/stroke (vs anoxia)	0.76	0.62 to 0.93	0.008
Head trauma (vs anoxia)	1.02	0.84 to 1.23	0.839
CNS tumor (vs anoxia)	0.13	0.02 to 0.90	0.039
Other (vs anoxia)	0.58	0.32 to 1.04	0.067
**CMV risk classification**			
Intermediate-risk group (vs low-risk group)	1.55	1.13 to 2.14	0.007
High-risk group (vs low-risk group)	1.50	1.04 to 2.16	0.031
Unknown-risk group (vs low-risk group)	4.14	2.97 to 5.77	<0.001

Abbreviation: IL-2: Interleukin 2; OKTAnti-CD3 antibody; CNI: Calcineurin inhibitor; HLA: Human Leukocyte Antigen; CNS: Central nerve system; CMV: Cytomegalovirus; 95%CI: 95% confidence interval
